# Attractiveness of the facial profile: comparison of Class II patients treated with Twin Force^®^ or intermaxillary elastics

**DOI:** 10.1590/2177-6709.26.5.e212014.oar

**Published:** 2021-10-15

**Authors:** Otávio Augusto POZZA, Rodrigo Hermont CANÇADO, Fabricio Pinelli VALARELLI, Karina Maria Salvatore FREITAS, Renata Cristina OLIVEIRA, Ricardo Cesar Gobbi de OLIVEIRA

**Affiliations:** 1 Centro Universitário Ingá - Uningá, Departamento de Ortodontia (Maringá/PR, Brazil).

**Keywords:** Malocclusion, Angle Class II, Comparative study, Esthetics

## Abstract

**Objective:**

To compare the facial profile attractiveness of Class II patients treated with Twin Force^®^ or intermaxillary elastics.

**Methods:**

Sample comprised 47 Class II patients divided into two groups: G1) TWIN FORCE - 25 patients treated with fixed appliances and Twin Force^®^ fixed functional appliance (mean initial age was 17.91 ± 7.13 years, mean final age was 20.45 ± 7.18 years, and mean treatment time was 2.53 ± 0.83 years); G2) ELASTICS - 22 patients treated with fixed appliances and Class II intermaxillary elastics (mean initial age was 15.87 ± 5.64 years, mean final age was 18.63 ± 5.79 years and mean treatment time was 2.75 ± 0.60 years). Lateral cephalograms from pretreatment and posttreatment were used. Cephalometric variables were measured and silhouettes of facial profile were constructed and evaluated by 48 laypeople and 63 orthodontists, rating the attractiveness from 0 (most unattractive profile) to 10 (most attractive profile). Intergroup comparisons were performed with Mann-Whitney and independent *t*-tests.

**Results:**

At pretreatment, facial profile of the Twin Force^®^ group was less attractive than the Elastics group. Treatment with Twin Force^®^ or Class II elastics resulted in similar facial profile attractiveness, but the facial convexity was more reduced in the Twin Force^®^ group. Orthodontists were more critical than laypeople.

**Conclusions:**

Treatment with Twin Force^®^ or Class II elastics produced similar facial profile attractiveness at posttreatment. Profile attractiveness was reduced with treatment in the elastic group, and improved in the Twin Force^®^ group. Facial convexity was more reduced with treatment in the Twin Force^®^ group.

## RESUMO

**Objetivo:**

Comparar a atratividade do perfil facial em pacientes Classe II tratados com Twin Force^®^ ou elásticos intermaxilares.

**Métodos:**

A amostra foi composta por 47 pacientes Classe II divididos em dois grupos: G1 - Twin Force^®^ (25 pacientes tratados com aparelhos fixos e o aparelho funcional Twin Force^®^; idade inicial média de 17,91 ± 7,13 anos, idade final média de 20,45 ± 7,18 anos, e tempo médio de tratamento de 2,53 ± 0,83 anos); G2 - Elásticos (22 pacientes tratados com aparelhos fixos e elásticos intermaxilares de Classe II, idade inicial média de 15,87 ± 5,64 anos, idade final média de 18,63 ± 5,79 anos, e tempo médio de tratamento de 2,75 ± 0,60 anos). Foram usadas telerradiografias laterais pré- e pós-tratamento. As variáveis cefalométricas foram mensuradas, e silhuetas do perfil facial foram construídas e avaliadas por 48 leigos e 63 ortodontistas, que pontuaram a atratividade entre 0 (perfil menos atraente) e 10 (perfil mais atraente). As comparações intergrupos foram realizadas com os testes Mann-Whitney e *t* de Student para amostras independentes.

**Resultados:**

Na fase pré-tratamento, o perfil facial no grupo Twin Force^®^ foi menos atrativo do que no grupo Elásticos. Os tratamentos com o Twin Force^®^ ou com Elásticos de Classe II resultaram em atratividade semelhante do perfil facial, mas a convexidade facial foi mais reduzida no grupo Twin Force^®^. Os ortodontistas foram mais críticos do que os leigos.

**Conclusões:**

Apesar de os tratamentos com o Twin Force^®^ ou com Elásticos de Classe II terem resultado em atratividade semelhante do perfil facial após o tratamento, a atratividade do perfil foi reduzida com o tratamento no grupo Elásticos e melhorou no grupo Twin Force^®^. A convexidade facial foi mais reduzida com o tratamento no grupo Twin Force^®^.

## INTRODUCTION

In the Class II treatment with intermaxillary elastics or fixed functional appliances, all skeletal and dentoalveolar changes produce effects on the soft tissue profile.^1^ ^-^ ^6^ Therefore, it is extremely important for the orthodontists to understand these effects to better perform the treatment planning and fulfill the esthetic expectation of each patient.

The interest in facial esthetics increases the search for orthodontic treatment; therefore, the modern orthodontics advances not only in the search for dental correction, but also at improving facial esthetics. The facial attractiveness is positively correlated with self-esteem, interpersonal and professional relationships.^7^

The appreciation of beauty is highly subjective.^8^ ^-^ ^11^ The attractiveness of the facial profile is a controversial subject in the literature, when comparing the perception of professionals and laypeople.^12^ Some studies show similar results among orthodontists and laypeople,^12^ ^-^ ^15^ while others show divergence of opinion.^11^ ^,^ ^16^ ^,^ ^17^ The satisfaction with facial and dental appearance is a predictor to know the patients’ expectations about orthodontic treatment.^18^

A previous study comparing the changes in profile attractiveness in children with Class II malocclusion treated with functional appliances and untreated subjects showed no difference, and the attractiveness was not improved with treatment.^19^ However, other studies with fixed and removable functional appliances showed improved facial profile attractiveness.^20^ ^-^ ^22^ Mendes et al.^23^ found similar attractiveness for nonextraction Class II treatment when compared to 2- and 4-premolar extraction. Janson et al.^1^ found similar soft tissue changes between Class II treatment with fixed functional appliances or maxillary premolars extraction.

Using cephalometric methods, the mandibular protraction appliance known as AdvanSync^®^was compared to intermaxillary elastics in the Class II treatment and both showed to be effective. AdvanSync^®^showed maxillary skeletal growth restriction and mandibular dentoalveolar changes, and Class II elastics showed only dentoalveolar changes.^24^ When compared to the Forsus^®^ mandibular protraction appliance, the Class II elastics showed similar treatment changes.^25^

Recent researches have indicated that orthodontic treatment with functional appliances is associated with increased facial profile attractiveness,^20^ ^,^ ^26^ ^,^ ^27^ and that functional treatment should be considered as a treatment option to improve the facial appearance of Class II subjects.^26^ Besides, Class II treatment with the Herbst appliance may produce a more esthetically improved facial profile silhouette when compared to the Forsus^®^ appliance, but the changes perceived by evaluators may not be considered clinically relevant.^27^

In the literature, no study comparing the attractiveness of the facial profile of patients with Class II malocclusion treated nonextraction with intermaxillary elastics or fixed functional appliances could be found. In this context, the present study aimed to compare the facial profile attractiveness in Class II patients treated with the functional fixed appliance Twin Force Bite Corrector^®^ or Class II intermaxillary elastics, evaluated by orthodontists and laypeople.

## MATERIAL AND METHODS

This study was approved by the Ethics in Human Research Committee of the Centro Universitário Ingá - Uningá (protocol 70881517.2.0000.5220).

The sample size calculation was based on an alpha significance level of 5% and a beta of 20% to achieve 80% of test power to detect a minimum difference of 0.88 points in the score of profile attractiveness, with a standard deviation of 1.02.^23^ Then, the sample size calculation showed the need of at least 22 subjects in each group (experimental and/or evaluators).

This retrospective study comprised 47 patients with initial Class II malocclusion treated with fixed appliances without extractions at the *Instituto Odontológico de Pós-graduação (IOPG, Bauru/SP, Brasil)*.

Inclusion criteria were: initial Class II malocclusion, treatment with fixed appliances without extractions, all teeth irrupted until first molars at the beginning of treatment, absence of agenesis or supernumerary teeth. Exclusion criteria were: patients that did not finish orthodontic treatment, who had their treatment plan changed due to lack of compliance, and no complete orthodontic records available. Selected patients were randomly divided into two groups: G1) treated with the Twin Force Bite Corrector^®^ functional appliance and G2) treated with Class II intermaxillary elastics.

Group 1 - TWIN FORCE: 25 patients (10 females, 15 males) orthodontically treated with fixed appliances and Twin Force Bite Corrector^®^ appliance (TFBC, Ortho Organizers, Inc, Carlsbad, CA, USA) for mandibular protraction. Mean initial age was 17.91 ± 7.13 years , mean final age was 20.45 ± 7.18 years, and mean treatment time was 2.53 ± 0.83 years.

Group 2 - ELASTICS: 22 patients (12 females, 10 males) treated with fixed appliances and Class II intermaxillary elastics. Mean initial age was 15.87 ± 5.64 years, mean final age was 18.63 ± 5.79 years and mean treatment time was 2.75 ± 0.60 years.

Patients of both groups were treated with preadjusted appliance (Roth prescription, Ortho Organizers, USA), with a similar archwire sequence: 0.014-in, 0.016-in and 0.018-in NiTi; 0.018-in, 0.020-in and 0.019 x 0.025-in stainless steel. When the rectangular wire was inserted, mechanics for Class II correction started. 

In Group 1, the Twin Force Bite Corrector^®^ (TFBC, Ortho Organizers Inc., Carlsbad, CA, USA) was installed and used for six to nine months. The TFBC is a fixed, intermaxillary functional appliance with ball-and-socket joint fasteners that allow a wide movement, including laterality.^28^ The appliance includes two telescopic tubes with NiTi coil springs that allows the delivery of a constant force.^28^

In Group 2, Class II intermaxillary elastics were used for 1 to 1.75 years. In both groups, Class II mechanics was used until a Class I molar and canine relationships were obtained.

Lateral cephalograms were evaluated in the initial (T_1_) and final (T_2_) stages of treatment. Cephalograms were scanned with the Microtek ScanMaker i800 scanner (Microtek International, Inc., Carson, CA, USA) with 9600 x 4800 dpi resolution. The images were transferred to the Dolphin Imaging Premium v. 10.5 software (Dolphin Imaging & Management Solutions, Chatsworth, CA, USA). The landmarks were digitized and the measurements were performed. The determination of the magnification factor of each device was performed, ranging from 6% to 10.2%, corrected by the software. Cephalometric variables included: SNA, SNB, ANB, Wits, 1.NA, 1-NA, 1.NB, IMPA, 1-NB, Overjet, Overbite and Facial Convexity (FC: angle formed by the intersection of the glabella-subnasale and subnasale-pogonion lines; G’.Sn.Pg’).

From the initial and final lateral cephalograms, silhouettes of facial profile were constructed with the CorelDRAW software (version 2017, Corel Corporation, Ottawa, Canada) and evaluated by orthodontists (Group A) and laypeople (Group B). 

All silhouettes were randomized for evaluation. In a Google^®^ forms questionnaire (LLC Google, Mountain View, CA, USA), sent by WhatsApp^®^ messenger app, the attractiveness of each profile silhouette was rated from 0 (most unattractive profile) to 10 (most attractive profile). The evaluators assessed the silhouettes for as long as needed, and were able to change the scores of attractiveness before submitting the form.^29^

Group A comprised 63 orthodontists (34 males and 29 females), with mean age of 39.91 ± 8.99 years - all individuals of this group were specialists in Orthodontics. Group B comprised 48 laypeople (31 males and 17 females), with mean age of 41.96 ± 12.52 years - laypeople were defined as individuals without formal education in dentistry or dental hygiene.

### ERROR STUDY

The reliability and precision of the methodology were verified by the Kappa coefficient in 20 randomly selected silhouettes, in which the attractiveness was reevaluated within a month interval. The Kappa coefficient was 0.85, considered an excellent agreement.^30^

After one month from the first measurements, 30 lateral cephalograms randomly selected were remeasured, and the intraclass correlation coefficient (ICC) was applied. All variables showed values of ICC above 0.9, indicating excellent agreement and reliability.

### STATISTICAL ANALYSIS

Normality of data was verified with Shapiro-Wilk test. Intergroup comparability of initial and final ages, treatment time and Little irregularity index was verified with independent *t*-test. Intergroup comparability of sex distribution and severity of the Class II malocclusion was verified by chi-square test.

Facial profile attractiveness was compared between the groups with Mann-Whitney test. Facial profile attractiveness was compared at pretreatment (T_1_) and posttreatment (T_2_), of patients treated with Twin Force^®^ or Class II elastics, by Wilcoxon test. Cephalometric variables at pretreatment (T_1_) and changes with treatment (T_2_-T_1_) were compared between the groups with independent *t-*tests.

All tests were performed with Statistica for Windows software (version 7.0; StatSoft, Tulsa, Oklahoma, USA), at *p*< 0.05.

**Figure 1:**
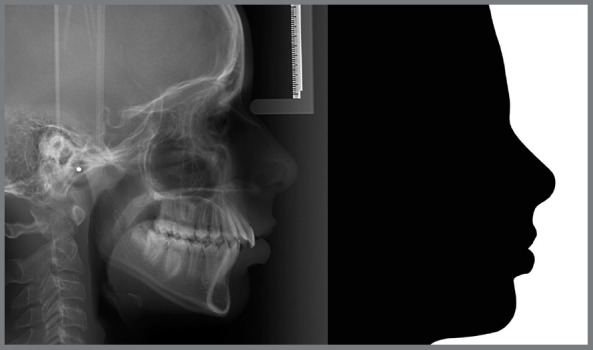
Example of a silhouette obtained from a lateral cephalogram.

## RESULTS

Groups 1 and 2 were comparable regarding initial and final ages, treatment time, Little irregularity index, sex distribution, severity of Class II malocclusion and pretreatment cephalometric variables (Tables 1, 2 and 3).

**Table 1:** Results of intergroup comparability of initial and final ages, treatment time, Little irregularity index, sex distribution and severity of Class II malocclusion.

Variables	GROUP 1 TWIN FORCE (n = 25)	GROUP 2 ELASTICS (n = 22)	*p*
	Mean ± SD	Mean ± SD	
Initial age (years)	17.91 ± 7.13	15.87 ± 5.64	0.2868^T^
Final age (years)	20.45 ± 7.18	18.63 ± 5.79	0.3480^T^
Treatment time (years)	2.53 ± 0.83	2.75 ± 0.60	0.3131^T^
Little irregularity index (mm)	5.28 ± 2.84	5.02 ± 3.28	0.7732^T^
SEX			X^2^ = 0.994
Male	15	10	DF = 1
Female	10	12	*p* = 0.3187^α^
Severity of Class II			
¼-cusp	0	1	X^2^ = 2.927
½-cusp	8	9	DF = 3
–-cusp	11	10	*p* = 0.4030^α^
Full cusp	6	2	

^T^ independent *t*-test; ^α^ chi-square test.

**Table 2:** Comparison of facial profile attractiveness at pretreatment (T_1_) and posttreatment (T_2_) between the groups 1 and 2 (Mann-Whitney test) and intragroup comparison of T_1_ x T_2_ (Wilcoxon test).

Variables	GROUP 1 TWIN FORCE n=25		GROUP 2 ELASTICS n=22		*p*
	Median (Mean)	IR (±SD)	Median (Mean)	IR (±SD)	
Facial profile attractiveness (T_1_)	5.00	3.00	6.00	3.00	0.000*^M^
	(4.65)	(± 2.60)	(5.41)	(± 2.26)	
Facial profile attractiveness (T_2_)	5.00	4.00	5.00	4.00	0.224^M^
	(4.98)	(± 2.35)	(5.06)	(± 2.42)	
*p*	0.000*^W^			0.000*^W^	

IR = interquartile range. SD = standard deviation. ^W^ Wilcoxon test. ^M^ Mann-Whitney test. * Statistically significant at *p*< 0.05.

**Table 3:** Results of the intergroup comparison of cephalometric variables at pretreatment (T_1_), and the treatment changes (T_2_-T_1_) (independent t-tests).

Cephalometrics Variables	GROUP 1 TWIN FORCE (n = 25)		GROUP 2 ELASTICS (n = 22)		*p*
	Mean	SD	Mean	SD	
INITIAL (T_1_)					
SNA (degrees)	82.25	± 3.18	82.96	± 3.46	0.466
SNB (degrees)	75.99	± 3.92	77.42	± 3.34	0.188
ANB (degrees)	6.27	± 2.09	5.53	± 1.94	0.215
Wits (mm)	5.56	± 2.25	4.79	± 2.31	0.257
1.NA (degrees)	21.28	± 8.93	21.69	± 11.18	0.891
1-NA (mm)	2.92	± 2.76	3.76	± 4.2	0.415
1.NB (degrees)	23.98	± 7.02	24	± 5.33	0.989
IMPA (degrees)	96.18	± 7.52	93.55	± 4.73	0.165
1-NB (mm)	4.4	± 2.43	4.45	± 2.24	0.946
Overjet (mm)	6.87	± 2.86	6.85	± 3.24	0.98
Overbite (mm)	4.51	± 1.73	4.03	± 2.39	0.431
Facial Convexity (degrees)	19.24	± 6.91	16.82	± 5.68	0.199
TREATMENT CHANGES (T_2_-T_1_)					
SNA (degrees)	-0.57	± 1.39	-0.38	± 2.19	0.721
SNB (degrees)	0.67	± 1.02	0.37	± 1.39	0.408
ANB (degrees)	-1.25	± 1.1	-0.74	± 1.43	0.177
Wits (mm)	-4.38	± 2.21	-3.91	± 2.09	0.456
1.NA (degrees)	-1.65	± 8.12	-1.04	± 10.37	0.821
1-NA (mm)	-0.68	± 2.33	-1.51	± 3.02	0.297
1.NB (degrees)	10.84	± 6.95	10.23	± 6.61	0.759
IMPA (degrees)	10.03	± 7.59	9.8	± 7.16	0.918
1-NB (mm)	2.2	± 1.99	1.7	± 1.44	0.345
Overjet (mm)	-4.11	± 2.72	-3.86	± 3.06	0.774
Overbite (mm)	-2.91	± 1.71	-2.47	± 2.31	0.46
Facial Convexity (degrees)	-3.07	± 3.52	-0.92	± 2.87	0.028*

* Statistically significant for *p*< 0.05.

At pretreatment, the Twin Force group presented a less attractive facial profile than the elastics group (Table 2). At posttreatment, facial profile attractiveness was similar between the groups (Table 2). In intragroup comparison of T_1_ and T_2_, the Twin Force^®^ group showed improvement of the facial profile attractiveness with treatment, and the Elastics group showed a reduction of the attractiveness of the profile (Table 2).

At pretreatment, the Twin Force^®^ and Elastics groups presented similar maxillomandibular skeletal discrepancy, incisor position, overjet, overbite and facial convexity (Table 3). Treatment changes of both groups were similar, except for the facial convexity that was more reduced in the Twin Force^®^ group than in Class II elastics group (Table 3).

The groups of orthodontists and laypeople were comparable regarding age and sex distribution (Table 4). Orthodontists were significantly more critical than laypeople in the evaluation of facial profile attractiveness at pre- and posttreatment (Table 5).

**Table 4:** Results of comparability of age and sex distribution between the groups of evaluators (A - Orthodontists and B - laypeople).

Variables	GROUP A Orthodontists (n = 63)	GROUP B Laypeople (n = 48)	P
	Mean ± SD	Mean ± SD	
Age (years)	39.91 ± 8.99	41.96 ± 12.52	0.3157^T^
SEX			X^2^=1.265 DF = 1 p = 0.2607^α^
Male	34	31	
Female	29	17	

^T^ independent *t*-test; ^α^ chi-square test.

**Table 5:** Comparison of facial profile attractiveness at pretreatment (T_1_) and posttreatment (T_2_) between orthodontists and laypeople (Mann-Whitney nonparametric test).

Variables	ORTHODONTISTS (n = 63)		LAYPEOPLE (n = 48)		P
	Median (Mean)	IR (±SD)	Median (Mean)	IR (±SD)	
Facial profile attractiveness (T_1_)	5.00	2.00	5.00	4.00	0.000*
	(4.92)	(±2.13)	(5.10)	(±2.58)	
Facial profile attractiveness (T_2_)	5.00	3.00	5.00	4.00	0.000*
	(4.86)	(±2.19)	(5.23)	(±2.61)	

IR = interquartile range. SD = standard deviation. * Statistically significant at *p* < 0.05.

## DISCUSSION

The interest in facial esthetics increased the search for orthodontic treatment and led orthodontists to seek treatments that result in better facial appearance. The esthetics of the facial profile can be evaluated in different ways, and the silhouette is a good method, since it eliminates confounding factors that influence the attractiveness, such as age, sex, skin, hair and eye color.^12^ ^,^ ^20^ ^,^ ^29^ ^,^ ^31^ Blinding of the evaluation stage of each silhouette was important, since the evaluators could be induced by the fact that the initial silhouettes were not treated, differently of the final ones.

In the literature, there is no known study comparing the attractiveness of the facial profile of Class II patients treated with fixed functional appliances or Class II intermaxillary elastics. Many authors have evaluated dentoalveolar and skeletal changes after treatment with mandibular protraction appliances^6^ ^,^ ^32^ ^-^ ^34^ or with Class II intermaxillary elastics.^3^ ^,^ ^35^ ^,^ ^36^ Some have compared these changes produced by Class II elastics and fixed or removable mandibular protraction appliances,^2^ ^,^ ^24^ ^,^ ^25^ ^,^ ^37^ however with little emphasis in the facial soft tissue profile changes. 

The initial ages and standard deviations of Groups 1 and 2 (Table 1) show that some patients were treated before and others after the pubertal growth peak. Yet, this finding occurred in both groups, and ages were comparable, with no impact on results. Besides, a previous study demonstrated that there is no difference in dentoskeletal effects after treatment with the Twin Force^®^ appliance prepubertal *vs* postpubertal patients with normodivergent pattern.^5^

The Twin Force^®^ and Elastics groups were comparable regarding initial and final ages, treatment time, mandibular anterior crowding, sex distribution, severity of Class II malocclusion and initial cephalometric characteristics (Tables 1 and 3). This evidence allows greater reliability in the comparison of attractiveness of the facial profile, minimizing possible differences in treatment effects.

Some residual growth may be present in some of the patients in both groups. However, since initial and final ages and treatment time were comparable between the groups, the possible residual growth changes would be similar in both groups, allowing a reliable comparison.

The Twin Force^®^ group presented a less attractive facial profile than the Elastics group at pretreatment stage (Table 2). This is probably because the Twin Force^®^ group presented a Class II slightly more severe than the elastics group, even though not significant statistically. The Twin Force^®^ group comprised 6 patients with full-cusp Class II molar relationship, and the Elastics group, only 2 patients (Table 1). This feature probably indicates a more convex and deficient facial profile in the Twin Force^®^ group, justifying the differences in the comparison of the pretreatment attractiveness between the groups. In cephalometric comparison of pretreatment stage, the facial convexity of the Twin Force^®^ group was greater, but without statistically significant difference from the elastics group (Table 3).

At the end of orthodontic treatment, there was no statistically significant difference of the facial profile attractiveness between the groups (Table 2). This outcome indicates that the facial profile attractiveness after treatment with the Twin Force^®^ mandibular protraction appliance and Class II elastics was similar. This finding corroborates the results of several studies evaluating and comparing the cephalometric effects of both treatment modalities. The studies found similar results of these therapies, indicating mainly dentoalveolar changes and minimal skeletal changes.^24^ ^,^ ^25^ ^,^ ^37^

In a systematic review of Class II correction with intermaxillary elastics, Janson et al.^3^ stated that the effects of this therapy are mainly dentoalveolar; little attention has been paid to the soft tissue effects, and long-term effects are similar to those produced by functional appliances.^3^

In intragroup comparison of pre and posttreatment stages, the Twin Force^®^ group showed a statistically significant improvement in facial profile attractiveness (Table 2). Since the Twin Force^®^ group presented a less attractive profile in the initial stage, and a slightly more convex profile and slightly greater mandibular retrusion, with no significant difference from the Elastics group, the use of the mandibular functional appliances was well indicated in these cases.^6^ ^,^ ^24^ ^,^ ^25^ With treatment, the profile convexity decreased and the facial profile attractiveness was improved, as already showed in previous studies evaluating fixed and removable functional appliances.^20^ ^,^ ^22^ ^,^ ^26^ ^,^ ^27^ ^,^ ^38^

However, the Elastics group showed a statistic significant reduction in the score of facial profile attractiveness with treatment (Table 2). The intergroup comparison of treatment changes showed that the facial convexity was more reduced in Twin Force^®^ than in elastics group (Table 3). In the elastics group, at pretreatment, the patients presented slightly smaller maxillomandibular discrepancy, indicating mainly dentoalveolar Class II problems, with less involvement of the facial profile, which may have resulted in a higher score of facial profile attractiveness, even observing in the cephalometric variables that there was a slight decrease in the facial convexity (Table 3). Treatment with Class II elastics can cause palatal inclination and retrusion of the maxillary incisors, and consequent retrusion of the upper lip, compromising the facial profile attractiveness.^3^ ^,^ ^39^ A previous study indicated that more prominent upper lips, less protruded lower lips, and more prominent chin might look more attractive.^39^

Regarding the evaluators, orthodontists were significantly more critical than laypeople in the evaluation of facial profile attractiveness at pretreatment and posttreatment stages (Table 4). This finding corroborates previous studies evaluating pre- and posttreatment silhouettes of orthodontically treated patients, which also found that orthodontists are more esthetically demanding than laypeople.^20^ ^,^ ^40^ ^,^ ^41^

These differences between orthodontists and laypeople could be justified because orthodontists have more knowledge regarding facial profiles, and the facial esthetic is related to straight and less convex profile.^20^ ^,^ ^27^

The perception of facial esthetics is not easy to understand and is highly subjective. The opinions of orthodontists, mostly in relation to dentofacial esthetics, take into consideration the ideal norms, guidelines and proportions, while the opinions of laypeople are motivated mainly by subjective feelings, such as culture of beauty and social norm of their environment.^42^ ^-^ ^44^

## CONCLUSION

Treatment with Twin Force^®^ or Class II elastics produced similar facial profile attractiveness at posttreatment. Profile attractiveness was reduced with treatment in the Elastics group, and improved in the Twin Force^®^ group. Facial convexity was more reduced with treatment in the Twin Force^®^ group.
